# The RNA N^6^-Methyladenosine MethylomeCoordinates Long Non-Coding RNAs to MediateCancer Drug Resistance by Activating PI3KSignaling

**DOI:** 10.21203/rs.3.rs-5663230/v1

**Published:** 2024-12-18

**Authors:** Shujun Liu

**Affiliations:** The Metrohealth System, Case Western Reser

## Abstract

Long non-coding RNAs (lncRNAs) and RNA N6-methyladenosine (m^6^A) have been linked to leukemia drug resistance. However, whether and how lncRNAs and m^6^A coordinately regulate resistance remain elusive. Here, we show that many differentially expressed lncRNAs enrich m^6^A, and more lncRNAs tend to have higher m^6^A content in CML cells resistant to tyrosine kinase inhibitors (TKIs). We demonstrate broad clinical relevance of our findings, showing that upregulation of top-ranked lncRNAs (e.g., SENCR, PROX1-AS1, LN892) in TKI resistant cell lines occurs in CML patients at the diagnostic stage, blast crisis phase or not-responding to TKIs compared to chronic phase or TKI responders, respectively. Higher lncRNAs predict drug resistance and shorter survival duration. Knockdown of SENCR, PROX1-AS1 or LN892 restores TKI sensitivity. Mechanistically, upregulation of PROX1-AS1, SENCR and LN892 results from FTO-dependent m^6^A hypomethylation that stabilizes lncRNA transcripts, and empowers resistant cell growth through overexpression of PI3K signaling mediators (e.g., ITGA2, F2R, COL6A1). Treatment with PI3K inhibitor alpelisib eradicates resistant cells *in vitro* and *in vivo* with prolonged survival of leukemic mice through downregulation of F2R, ITGA2 and COL6A1. Thus, the lncRNA-m^6^A-PI3K cascade represents a new non-genetic predictor for drug resistance and poorer prognosis in cancer, and a pan-cancer mechanism underlying TKI resistance.

## Introduction

The constitutively activated tyrosine kinases (TKs) are hallmark features of several types of cancer, such as chronic myeloid leukemia (CML) and Ph + ALL. It is well known that the growth and progression of chronic myeloid leukemia (CML) is attributed to a hybrid protein BCR::ABL1 1,(1) resulting from the t(9;22)(q34;q11) chromosomal translocation. Multiple TK inhibitors (TKIs), such as imatinib, nilotinib, dasatinib, bosutinib and ponatinib, have emerged as leading compounds to treat CML. Initial clinical response of CML patients to TKIs is often excellent.(2–5) However, relapse and disease progression characterized by drug resistance occurs even with continuous drug administration, and patients with TKI resistant CML may still succumb to their disease.(6–12) Studies regarding TKI resistance in leukemia have mostly centered on cancer genetic alterations,(13, 14) for example, acquired mutations (which happen in about 1/3 of progressive cases) in the kinase domains of BCR::ABL1 that reduce or even fully impair TKIs’ binding to ABL kinase. However, it is less known whether and how dynamic and reversible RNA epitranscriptomic mechanisms play a role in TKI resistance, particularly for resistant patients without acquired mutations. In the current work, we tested the hypothesis that epitranscriptomic diversity in heterogeneous tumor cell populations may generate divergence in the expression of cell fate determination genes/pathways that can swiftly avoid drug-induced cell death.

*N* ^*6*^ -methyladenosine (m^6^A) is the most common internal modification on RNAs, including long non-coding RNAs (lncRNAs). The m^6^A is installed by a methyltransferase complex,(15, 16) erased by the demethylases (e.g., FTO),(17–20) and recognized by m^6^A-binding proteins. Cumulative evidence suggests that m^6^A methylation occurs frequently in a dynamic and reversible manner,(21–26) and significantly regulates RNA half-life, RNA splicing and protein translation,(27–30) thus determining target expression levels. Defects in m^6^A methylation affect diverse biological processes, including cancer initiation, development and progression.(31–35) We recently demonstrated that a dynamic FTO/m^6^A axis emerges as a key epigenetic driver of reversible TKI-tolerance state making contribution to acquired TKI resistance through activating cell proliferation/anti-apoptotic genes.(36) Yet, it is not well understood whether and how the FTO/m^6^A axis empowers leukemia cells to survive prolonged drug exposure through non-protein coding genes.

LncRNAs are transcripts with lengths of ≥ 200 nucleotides and have no apparent protein-coding potential. As they crucially activate or repress transcription of protein-coding genes,(37) lncRNA dysregulations have been linked to cancer progression and drug resistance.(38–46) Previous studies suggest that functions of lncRNAs are subjected to m^6^A regulation through, for example, potentiating lncRNAs-mediated transcriptional repression.(47, 48) These findings, together with the observations that upregulation of certain lncRNAs fuels the survival and proliferation of cancer cells,(49–51) raise the possibility that, upon exposure to TKIs, the dynamic m^6^A methylation allows a set of proliferation/anti-apoptosis-relevant lncRNAs bearing m^6^A motifs to be rapidly upregulated, thus helping a subpopulation of cells avoid TKI killing. In the current work, we examined the potential interactions between m^6^A methylome and lncRNAs in resistant leukemia cells and found that this interaction shapes the balance between TKI resistance and sensitivity. We dissected its pathological relevance in leukemia patients and found that m^6^A-upregulated lncRNAs are novel biomarkers associated with TKI non-responders and predict worse outcomes.

## Materials and Methods

### Mice

All animal experiments were approved by the Institutional Animal Care and Use Committees of the University of Minnesota and were in accordance with the US National Institutes of Health Guide for Care and Use of Laboratory Animals. For survival studies, mice were sacrificed when they showed any signs of distress (i.e., breathing disorders, weight loss or immobility).

The NOD/SCID/γcnull immunodeficient NOG mice (female, male; 4–6 weeks old) were purchased from Charles River and sublethally irradiated. About 0.5 × 10^6^ nilotinib resistant K562 cells were injected into the irradiated mice through the tail vein 4 hours after irradiation. Recipient mice were monitored weekly for signs of leukemia beginning on day 7 after transplantation. Mononuclear cells collected from bone marrow (BM) of the euthanatized mice were stained with mouse anti-human CD45 antibody (Bioligned) for flow cytometry sorting. The presence of a CD45+ population was considered as human leukemia engraftment. The development of leukemic disease was verified by H&E staining. Then BM cells were isolated for further investigations.

For preclinical testing of PI3K inhibitor: Alpelisib was prepared just prior to administration by dissolving in DMSO to provide a clear solution and then diluting with 2-Hydroxypropyl-β-cyclodextrin (HPCD) and PBS (ratio 2.5:75:22.5). About 0.5 × 10^6^ BM cells isolated from the aforementioned leukemic mice will be injected into the sublethally irradiated NSGS mice (second recipient). The leukemia-bearing mice were randomized and administrated alpelisib intraperitoneally every three days at 100 mg/kg for 5 weeks. The administration of vehicle (DMSO, HPCD, PBS; ratio 2.5:75:22.5) was used as a negative control. The survival time was analyzed by Kaplan–Meier estimate, and comparison between groups was analyzed by log-rank tests. The survival time was from the start of leukemia cell injection. Cytospin preparations of BM cells were processed for Wright-Giemsa staining. The lungs, spleens, and livers were immediately fixed in 10% neutral-buffered formalin and stained with H&E.

### AML and CML patient samples

The current study was approved by the Institutional Review Board of Mayo Clinic and the second hospital of Shanxi Medical University and conducted in accordance with the Declaration of Helsinki. The diagnoses of acute myeloid leukemia (AML) and chronic myeloid leukemia (CML) were made according to the criteria of World Health Organization. Mononuclear cells from BM or PB samples of AML and CML patients were prepared by Ficoll-Hypaque (GE Healthcare #71–7167-00) gradient centrifugation. The patient cells were frozen in 10% DMSO plus 90% FBS and directly used for molecular biological assays without further cell culture. All patients signed an informed consent document approved by the Institutional Review Board before entering any study.

### Cell lines and cell culture

Leukemia cell lines, K562, MV4–11 and Kasumi-1, were newly purchased from American Type Culture Collection with no further authentication or testing for mycoplasma. Cell lines were grown in RPMI-1640 (GE Healthcare #SH30027.01) supplemented with 20% (Kasumi-1) or 10% (K562, MV4–11) fetal bovine serum (FBS, Gibco by Life Technologies^™^ #16140–071) and Antibiotic-Antimycotic (Gibco by Life Technologies^™^ #15240062) at 37°C under 5% CO_2_. No cell line used in this paper is listed in the database of commonly misidentified cell lines maintained by ICLAC (International Cell Line Authentication Committee).

### Plasmid design and construction

Three shRNAs against *FTO* (TRCN0000183897, TRCN0000179651, TRCN0000180978) and the negative control vectors (pLKO.1) were obtained from BMGC RNAi (University of Minnesota), but the shRNA against lncRNAs PROX1-AS1 (NR_037850.2; AS7, AS1521, AS3299), SENCR (NR_038908.1; S16, S785, S1238) and LN892 (NR_038461.1; LN370, LN619, LN576) were designed using the online tool (https://portals.broadinstitute.org/gpp/public/seq/search), synthesis by Integrated DNA Technologies Inc, and cloned into pLKO.1 vector. The primers are listed in Supplementary Table 7.

### *In vitro* adaption of TKI resistant cells

Cell lines, K562, Kasumi-1 and MV4–11, were passaged with low concentration of imatinib or nilotinib (0.1 μM) and sequentially cultured in increasing concentrations of these TKIs (0.3, 1 μM) for 8–10 weeks. Cells cultured in parallel in drug-free medium were used as parental/sensitive controls. Cells were considered resistant when they could routinely grow in medium containing 1 μM imatinib, or nilotinib, respectively.

### Lentivirus vector, virus production and virus infection

For virus production, HEK-293 (3.8 × 10^6^) cells were planted in a 10 cm cell culture dish for 24 hours, and transfected with 6 μg of target or scrambled control plasmids using calcium phosphate transfection reagent (CalPhos^™^ Mammalian Transfection Kit), following the manufacture’s instruction. The lentiviruses were harvested at 48 and 72 hours after transfection and concentrated using the protocol of the Lenti-X^™^ Concentrator (Clotech #631232). For virus infection, leukemia cells (1 × 10^6^) were infected by the lentiviruses using Polybrene (final concentrate 4 μg/ml) in 1ml medium and Puromycin (final concentration 2 μg/ml) was added to select the stable transformants 24 hours post-infection.

### Colony-forming assays

Colony-forming assays were performed using MethoCult^®^ medium (Stem Cell Technologies #03434) as previously reported.(36,52,53) Briefly, the cells were suspended in 0.3mL of IMDM medium (Stem Cell Technologies #36150), mixed with 3 ml MethoCult^®^ medium and then dispensed into 35 mm dishes. The colony count and size were recorded after 7–10 days. Generally, 10 single clones were picked up into 96 well plate per each cell line in order to get the stable clone after virus infection and puromycin selection.

### m^6^A Dotblotting

The mRNA was extracted using GenElute^™^ direct mRNA Miniprep Kit (Sigma #DMN70–1KT). The m^6^A RNA dotblotting was performed with a Bio-Dot Apparatus (Bio-Rad #170–6545). About 500 ng mRNA was diluted in 50 μl RNase-free water and mixed with 150 μl RNA incubation solution (1 ml mix: 657 μl formamide, 210 μl 37% formaldehyde solution and 133 μl 10 × MOPS). Then the RNA was denatured at 65°C for 10 min and mixed with 200 μl ice-cold 20 × SSC. The RNA loading membrane was baked at 80°C for 5 min, UV crosslinked, blocked with 5% non-fat milk at room temperature for 45 min, and incubated with m^6^A antibody for overnight. After being washed for 3 times with 1 × PBST, the membranes were incubated with a HRP-conjugated secondary antibody anti-rabbit in 5% non-fat dry milk. The signal was detected by enhanced chemiluminescence. RNA spotted membrane was stained with 0.02% methylene blue (Sigma #1808) in 0.5 M sodium acetate (pH 5.0) for loading control. The antibodies used are listed in Supplementary Table 6.

### Western Blotting

The whole cellular lysates were prepared by harvesting the cells in 1 × cell lysis buffer [20 mM HEPES (pH 7.0), 150 mM NaCl and 0.1% NP40] supplemented with 1 mM phenylmethane sulfonyl fluoride (PMSF, Sigma #10837091001), 1 × Phosphatase Inhibitor Cocktail 2 and 3 (Sigma #P5726, P0044), and 1 × protease inhibitors (protease inhibitor cocktail set III, Calbiochem-Novabiochem #539134). The proteins were resolved by sodium dodecyl sulfate (SDS)–polyacrylamide gel electrophoresis, transferred onto PVDF membranes (GE Healthcare #10600023), blocked by 5% non-fat milk followed by probing with first and HRP-conjugated secondary antibodies (listed in Supplementary Table 6).

### RNA isolation, cDNA preparation and quantitative PCR (pPCR)

According to manufacturers’ instructions, the total RNA was isolated using miRNeasy Kit (Qiaqen #217004), and complementary DNA (cDNA) synthesis was performed using SuperScript^®^ III First-Strand Synthesis System (Invitrogen #18080–051). The expression of target genes was assessed by SYBR Green qPCR (Applied Biosystems #4309155). The expression of the targets was analyzed using the DCT approach. The levels of *GAPDH or 18s* were used as normalization in cell lines, but ABL was used as internal control in patient sample according to BCR::ABL1 IS. The primers are listed in Supplementary Table 7.

### m^6^A immunoprecipitation (IP)

The RNAs were diluted in 200 μl IPP buffer (150 mM NaCl, 0.1% NP-40, 10 mM Tris-HCl, pH 7.4) and fragmented into 100-nucleotide-long fragments using sonication. The fragmented RNAs were incubated for 12 hours at 4 °C with 5 μl anti-m^6^A (listed in Supplementary Table 6) in IPP buffer. The mixture was then immunoprecipitated by incubation with Dynabeads^™^ Protein G (ThermoFisher #10004D) at 4 °C for additional 3 hours. After extensive washing by IPP buffer, 75 μl 42°C pre-heated Elution Buffer (0.02 M DTT, 0.15 M NaCl, 0.05 M Tris-HCl, pH 7.4, 0.001 M EDTA, 0.1% SDS) were added into m^6^A-positiveRNA solution for 5 min at 42°C, and this step was repeated 2 times. Finally, the enriched m^6^A RNA was eluted from the beads into 225 μl solution and precipitated by adding 2.5 times volume of 100% ethanol.

### Cell proliferation and apoptosis assays

Cell proliferation assays were performed using Cell Counting Kit-8 (CCK-8, Dojindo Molecular Technologies #CK04–11) following manufactures’ instruction. Briefly, the parental and resistant cells with various treatment (1.5 × 10^4^) in RPMI-1640 medium (100 μl) were dispensed into 96-well flat-bottomed microplates and incubated for 24 hours. The cells were cultured for another 24 or 48 hours, and CCK-8 reagent (10 μl) was added to each well. The microplates were incubated at 37°C for additional 2~4 hours. Absorbance was read at 450 nm using a microplate reader and the results were expressed as a ratio of the treated over untreated cells (as 100%). Five wells were sampled per each experimental group in a given experiment. Averages are reported ±SD. Cell apoptosis assays were performed using Annexin V-PI Apoptosis Detection Kit I (BD Pharmingen^™^ #556547) according to the manufacturer’s instruction, and followed by flow cytometry analysis.

### Identification of differentially expressed annotated lncRNAs

For all IP and IN samples, the RNA-seq raw reads were aligned to the UCSC human reference genome (GRCh38/hg38) by Hisat2(54) with parameter –U and then sorted and indexed by SAMtools.(55) The mapped reads were counted using featureCounts v1.6.1(56) and a differential expression analysis comparing resistance and parental samples was performed using R packages, edgeR(57) and limma. (58) The differentially expressed lncRNAs (annotated by GENCODE v19) with an absolute value of log_2_FC>0 and FDR < 0.05 were considered for future analysis. Top 40 most differential expressed LncRNAs having m^6^A sites in nilotinib samples were selected from the upregulated lncRNAs and a heatmap of these lncRNAs ranked by log_2_FC in a decreasing order was plotted by R package pheatmap.

### Peak reads count analysis for m^6^A sequencing

Peaks were called for each of the three groups by using MeTPeak R package as described previously and were further filtered by choosing only those assigned to annotated lncRNAs. For lncRNAs that have more than one peaks, the mean of fold enrichment of all its peaks was used as its fold enrichment. To compare m^6^A enrichment of lncRNAs between resistant and parental samples, the lncRNAs occurred only in one sample were assigned 0-fold enrichment in the other sample, and then log_2_ (resistant fold enrichment) – log_2_ (parental fold enrichment) was calculated to obtain log_2_ Fold Change (if one of these two-fold enrichment values is 0, add 1 to both before log_2_ transformation), finally, R package ggplot2 was used to plot a histogram for the log_2_ Fold Change calculated above.

### Hematoxylin & eosin (H&E) staining

Mouse tissues (lung, liver, spleen) were fixed in 10% neutral-buffered formalin, deparaffinized, hydrated, and stained with H&E (Thermo-Scientific) staining was performed at the image center, the MetroHealth System, Case Western Reserve University.

### Cytospin/Wright-Giemsa staining

The mouse BM cells (0.1 × 10^6^) were isolated and placed in the Shandon EZ Single Cytofunnel (Thermo Electron Corporation). Samples were centrifuged at 1,000 rpm for 8 min. The slides were air-dried and stained with Hema-3 Kit (22–122-911, Fisher Scientific, Hampton, NH). Stained slides were viewed and photographed using a Leica microscope mounted with a high-resolution spot camera with Image-Pro Plus software. Morphologic differentiation was determined by calculating the percentage of post-mitotic cells containing metamyelocytes, bands and segmented neutrophils within six visual fields per slide (original magnification × 200).

### Analysis of gene expression omnibus (GEO) data and functional pathways

LncRNA and gene expression profile data were downloaded, and analyzed for the expression of lncRNAs. These samples were normalized, managed and analyzed by GraphPad Prism 5 Software. Further, pathway and gene-enrichment analyses that are associated with SENCR expression were conducted using DAVID 6.8 (KEGG_pathway) software. The significance of the association between target genes and canonical pathway was computed using two parameters: 1) a ratio of the number of target genes from the dataset that map to the pathway divided by the total number of genes that constitute the canonical pathway; and 2) a −log_10_ (*P*-value) determining the probability that the association between the DEGs in the dataset and the canonical pathway is due to chance alone. In the present analysis, the computed −log_10_ (p-value) of 3.0 (*P* < 0.05) and above was considered as statistically significant.

### Analysis of MLL database

The cohort comprised a total of 985 patients: 64 healthy individuals, 774 AML patients, and 121 CML patients at initial diagnosis. Furthermore, sequential samples of 12 CML patients with progression from chronic to blast phase CML were analyzed. Bone marrow or peripheral blood (PB) samples from these patients had been sent to MLL Leukemia Laboratory between 2006 and 2023 for diagnostic work-up. The respective diagnosis was established based on cytomorphology, immunophenotyping, cytogenetics, and molecular genetics following WHO guidelines. AML patients were grouped according to European Leukemia Net (ELN) 2022 risk group.(59)All patients gave their written informed consent for scientific evaluations. The study was approved by the Internal Review Board and adhered to the tenets of the Declaration of Helsinki. RNA was extracted using the MagNA Pure 96 Cellular RNA LV Kit (Roche LifeScience, Mannheim, Germany). For transcriptome analysis the TruSeq Total Stranded RNA kit was used, starting with 250ng of total RNA, to generate RNA libraries following the manufacturer’s recommendations (Illumina, San Diego, CA, USA). 2 × 100bp paired-end reads were sequenced on the NovaSeq 6000 (Illumina, San Diego, CA, USA) with a median of 50 million reads per sample. Using BaseSpace’s RNA-seq Alignment app (v2.0.1) with default parameters reads were mapped with STAR aligner (v2.5.0a) to the human reference genome hg19 (RefSeq annotation). Estimated read counts for each gene were normalized applying Trimmed mean of M-values (TMM) normalization method and the resulting log2 counts per million (CPMs) were used.

### Statistical analysis

The statistical analysis was performed using the Student’s *t* test. All analyses were performed using the GraphPad Prism 5 Software. *P* < 0.05 was considered statistically significant. All *p* values were two-tailed. No blinding or randomization was used. No samples or animals were excluded from analysis. All criteria were pre-established. No statistical method was used to predetermine sample size and the sample size for all experiments was not chosen with consideration of adequate power to detect a pre-specified effect size. Variations were compatible between groups. *In vitro* experiments, such as qPCR, Western blotting, cell proliferation assays, dotblotting etc. were routinely repeated three times unless indicated otherwise in Figure legends or main text. For every Figure, the statistical tests were justified as appropriate.

## Results

### The activities of the targeted kinase signaling are not required for the survival and proliferation of TKI-resistant cells

**Figure 1:**
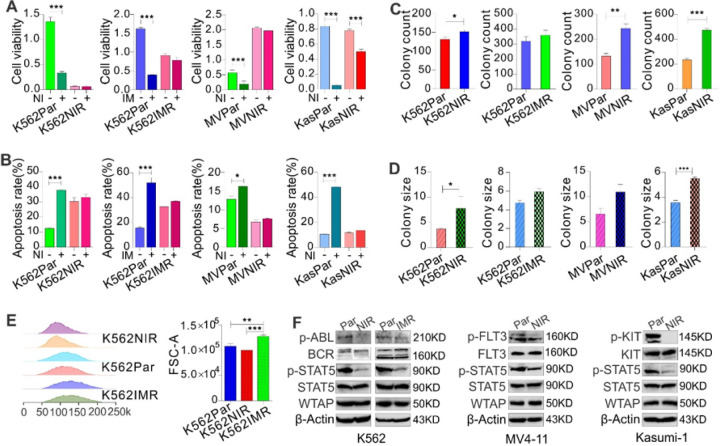
Generation and characterization of leukemia cells with acquired resistance to nilotinib or imatinib. **(A-B)** CCK-8 assays for cell proliferation and flow cytometry assays for cell apoptosis in parental and resistant cells treated with 1 μM nilotinib or imatinib for 72 hours. The data of CCK-8 represent twoindependent experiments with 6 repeats in total, but flow cytometry assays represent three independent experiments. **(C-D)**Colony-forming assays for K562, Kasumi-1 and MV4–11 parental and resistant cells in drug free medium. **(E)** Flow cytometry assays to measure cell size in K562 resistant and parental cells. **(F)** Western blotting of parental and resistant cells growing in drug-containing medium. In Figure **A-E**, data are expressed as mean values ± S.E.M. of triplicate samples. In Figure **F**, the data are representative of 3 independent experiments. The figures were made by cropping the large gel or blot images. Par, parental; NIR, nilotinib resistant; IMR, imatinib resistant; NI, nilotinib; IM, imatinib; MV, MV4–11; Kas, Kasumi-1. *P < 0.05, **P < 0.01, ***P < 0.001.

To dissect the molecular mechanisms of acquired resistance to inhibitors of TK pathway, we generated drug-resistant derivatives of leukemia cells K562 (BCR::ABL1 ), MV4–11 (FLT3) and Kasumi-1 (c-KIT) by long-term culture in the presence of TKIs nilotinib (second generation) or imatinib (first generation) (10, 30, 100, 300, 1,000 nM). Cells cultured in parallel without drugs serve as parental/sensitive controls. Cells are considered resistant when they can routinely grow in medium containing 1 μM nilotinib or imatinib, respectively. We then characterized the resistant phenotypes by measuring the survival rate of parental and resistant cells upon transient exposure to TKIs. All parental controls displayed a significant decrease in cell viability (Figure 1A) and an increase in apoptotic cells (Figure 1B; Figure S1a), but the resistant cells could proliferate in the drugs with no obvious changes of cell apoptosis. Results from colony-forming assays were heterogeneous (Figure 1C and D; Figure S1b), in which nilotinib-resistant K562, Kasumi-1 and MV4–11 cells have significantly higher colony-forming potential with more colonies and larger colony sizes. However, imatinib-resistant K562 cells did not show significant changes in colony number and size compared with parental cells. Flow cytometry detected different cell sizes between parental and resistant K562 cells, in which imatinib resistant cells are the largest (Figure 1E). Finally, we examined the activities of the targeted TKs, and found that, compared to parental cells, the phosphorylation of KIT, FLT3 and BCR::ABL1 is decreased without obvious changes of total protein expression when growing in drug-containing medium, leading to dephosphorylation of STAT5 (Figure 1F), a shared downstream signaling mediator. These results suggest that survival and proliferation of TKI resistant cells are independent of the targeted TK signaling.

### Transcriptome-wide m^6^A sequencing unveils distinct lncRNAs in nilotinib resistant versus sensitive leukemia cells

**Figure 2:**
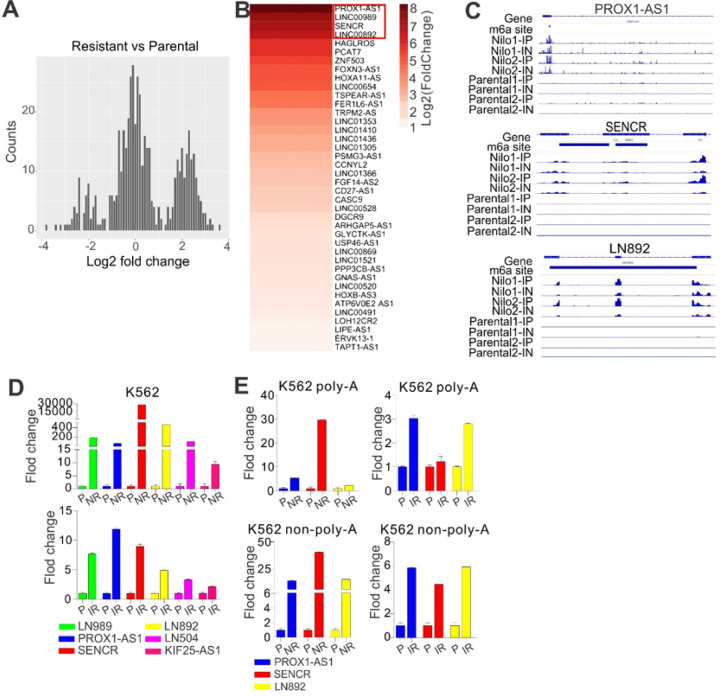
Differentially expressed lncRNAs in nilotinib resistant cells bear m^6^A motifs. **(A)** Plot distribution of log_2_ change of enrichment across m^6^A peaks from parental to nilotinib resistant cells. The average log_2_-transformed normalized signal for the duplicated m^6^A-seq was used to generate a histogram of read counts values. **(B)** The heatmap based on the sorted log_2_-fold change of 40 upregulated lncRNAs. Fourty differentially expressed lncRNAs (annotated by gencode v19) are ranked by log_2_ (fold change) with different m^6^A methylation levels. **C** lncRNAs PROX1-AS1, SENCR and LN892 with m^6^A-seq track are highlighted. **(D)** qPCR of total RNA for the expression of indicated lncRNAs in parental and resistant K562 cells. **(E)** qPCR for lncRNA expression in poly-A and non poly-A RNA of K562 P, IR and NR cells. Data are expressed as mean values ± S.E.M. of duiplicate samples from three independent experiments. P, parental; NR, nilotinib resistant; IR, imatinib resistant; IP, immunoprecipitationl; IN, Input; Nilo, nilotinib.

Our previous studies(36) showed that, compared with parental cells, most changes of m^6^A peaks in resistant cells come from lncRNAs as well as CDs and 3’UTR with less profound changes at 5’UTR. These findings imply that m^6^A-associted lncRNAs may partially affect the development of TKI resistance. To test this, first we analyzed the m^6^A-peak fold enrichment from normalized read counts mapped to peak region focusing on the changes of lncRNAs. We found that the distribution of log_2_ transformed fold change show three peaks, the highest peak in the middle part of the distribution indicating that most lncRNAs have comparable m^6^A levels with log_2_ fold change close to 0, while the peaks of the positive log_2_ fold change located on the right side of the distribution are much higher than those of the negative log_2_ fold change on the left side, indicating that relative more lncRNAs tend to have higher m^6^A in resistant cells (Figure 2A). These observations suggest that, compared with parental cells, the overall m^6^A peak fold enrichment is decreased,(36) but lncRNAs, in general, have a trend of higher m^6^A content in resistant cells. Second, we analyzed m^6^A RNA sequencing data,(36) and identified 315 upregulated and 97 downregulated lncRNAs (annotated; Supplementary table 1) in resistant versus parental cells. By intersection analyses of differentially expressed lncRNAs with changed m^6^A peaks (Figure S2a), we identified 40 upregulated lncRNAs bearing m^6^A sites in Nil (nilotinib resistant) samples (Supplementary table 2). The heatmap in Figure 2B was plotted based on the sorted log_2_-fold change of top 40 overlapped lncRNAs. For all immunoprecipitation (IP) and input (IN) samples, the sorted bam files along with m^6^A site coordinate information stored in bed files were loaded into Integrative Genomics Viewer (IGV) to compare expression levels of interested intergenic lncRNAs (e.g., LN892) across different conditions (Figure 2C).

We conducted qPCR for total RNA from K562 cells, and verified the alterations of top-ranked lncRNAs (Figure 2C; Figure S2b), for example, upregulation of LN989, PROX1-AS1, SENCR, LN892, LN504, and KIF25-AS1; downregulation of LN659, VPS9D1-AS1, LNC200, UCA1 and WASIR2; but no change in LN1270 and MAP3K14-AS1. Given that our RNA-seq is done in the fraction of poly-A-associated RNAs, and as lncRNAs have both poly-A and non-poly-A tailed RNAs,(60) we separated poly-A tailed RNAs from non-poly-A tailed RNAs, and measured the lncRNA levels. We observed that the expression of PROX1-AS1, LN892 and SENCR is upregulated as a similar pattern in both poly-A and non-poly-A tailed fraction (Figure 2D). Pathway analysis (Figure S3) revealed that PROX1-AS1 regulated pathways are mostly negatively enriched, but LN892 and SENCR regulated pathways are mostly positively enriched; pathways in red boxes are positively enriched pathways regulated by LN892 and SENCR; MYC pathway in blue box is significantly negatively enriched and regulated by all three lncRNAs; PI3K-AKT/mTOR signaling is negatively enriched and regulated by PROX1-AS1 but has trends toward being enriched when regulated by SENCR and LN892. Taken together, these data suggest that m^6^A-associated lncRNAs are involved in a non-genetic mechanism that underlies the emergence and maintenance of acquired TKI resistance.

### Expression of the m^6^A-associated lncRNAs is particularly elevated in leukemia patients at diagnosis or not-responding to TKIs, and could serve as an independent predictor for poor prognosis

**Figure 3:**
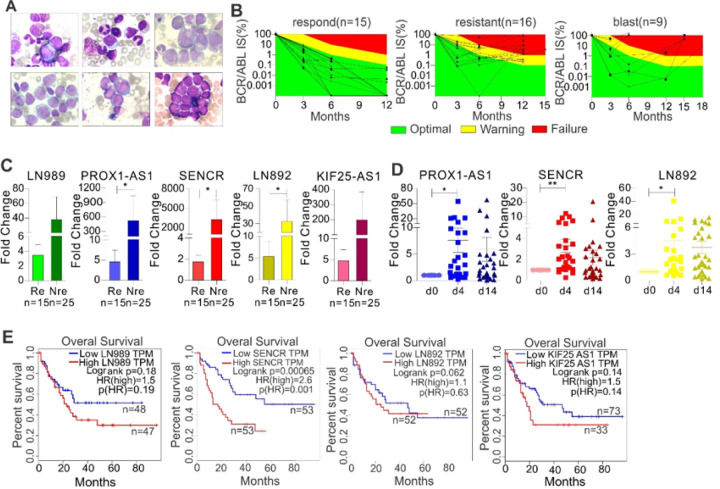
lncRNA upregulation predicts unfavorable outcomes in leukemia patients. **(A)** BM cell morphology of CML patients. The classical morphological images were obtained from some CML-CP and CML-BP patients. Bone marrow cells were cytospinned onto slides followed by Wright-Giemsa staining 20 minutes. The images were obtained under the light microscope. Upper, the chronic phase; lower, The blast phase (AML and ALL). **(B)**Changes in the number of BCR::ABL1 transcripts prior and post nilotinib or imatinib therapy. Green, yellow and red areas represent optimal, warning and failure results, respectively. **(C)** qPCR for lncRNA expression in CML (n = 40) patients who are classified as responder and inadequate responders. Median values are depicted by the horizontal lines. **(D)** qPCR of RNA extracted from AML patients (n = 30) receiving nilotinib therapy. **(E)** The association of lncRNA expression with overall survival (OS) in leukemia patients analyzed by the Kaplan–Meier estimate. Re, Responder; Nre, Non-responder. *P < 0.05, **P < 0.01.

To explore the clinical implications of the dysregulated lncRNAs, first, we analyzed the gene expression profiling of AML collected at diagnosis, and compared the expression of FTO, LIN892, LIN989, PROX1-AS1 and SENCR among different response groups. In these AML patients, a trend towards higher levels of expression of LIN989 and SENCR was observed in the ELN risk groups intermediated and adverse (Figure S4a). In line, expression of SENCR at diagnosis was higher in AML patients that did not achieve complete remission (CR) after induction chemotherapy (Figure S4b). Furthermore, we performed an exploratory analysis of matched samples from CML patients at diagnosis in chronic phase, during hematologic remission and at the time of progression to blast phase CML (Figure S4c). Expression of FTO was highest at blast phase and lowest at hematologic remission. Also, differential expression of lncRNAs was overserved in this cohort. Such heterogeneous correlation between the expression of given genes and drug responses could be attributed to the variations of blast percentage, genetic mutations, age and sex, which will be validated in a larger cohorts of patients. To further clarify the role of FTO, LIN892, LIN989, PROX1-AS1 and SENCR in drug resistance, we obtained CML PB cells from patients after nilotinib or imatinib therapy. The patient characteristics are described in Supplementary table 3. The definition of responding and inadequately responding was based on the number of BCR::ABL1 transcripts post therapy, but the chronic phase (Figure 3A, upper panel; n = 3) versus blast crisis (Figure 3A, lower panel; n = 3) was determined by the cell morphology and relative clinical information. When the BCR::ABL1 international score (IS) meets response milestones at 3, 6, and 12 months (≤ 10% BCR::ABL1 IS at 3 and, ≤ 1% BCR::ABL1 IS at 6 months and ≤ 0.1% BCR::ABL1 IS at ≥12 months) post therapy, these patients are classified as TKI responders, and others as inadequate responders (Figure 3B).(61) We then performed qPCR in normal and CML patient cells for LN989, PROX1-AS1, SENCR, LN892 and KIF25-AS1, which are consistently elevated in resistant K562 cells. All aforementioned lncRNAs are significantly upregulated in CML patients when compared with normal blood cells (Figure S4d). We also employed online tools GEPIA(62) and BloodSpot(63) to further validate the expression patterns of the aforementioned lncRNAs in leukemia patients and normal donors. Consistently, expression of LN892 and SENCR in GEPIA as well as PROX1-AS1 and KIF25-AS1 in Bloodspot (Leukemia MILE Study) is much higher in leukemia patients than those in normal donors (Figure S5a, S5b) with barely or undetectable expression of LN892 in both datasets. When compared with TKI responders, CML patients with inadequate response had significantly higher expression of LN989, PROX1-AS1, SENCR, LN892, and KIF25-AS1 (Figure 3C). When compared with chronic disease, patients with blast crisis had a trend toward higher lncRNA expression (Figure S5c, S5d). Notably, to strengthen the conclusion, we employed both 18S and ABL as internal controls to normalize lncRNA expression in patients. The same conclusions were made when using ABL, the most cited internal control, although more obvious difference was observed when 18S was used (Figure S5e).

To further substantiate the clinical significance of the m^6^A-assicated lncRNAs in leukemia, we also examined the expression of LN989, PROX1-AS1, SENCR, LN892 and KIF25-AS1 in AML patients, who received nilotinib twice daily after induction and consolidation chemotherapy.(36) We observed that nilotinib (in combination with chemotherapy) upregulates LN989, PROX1-AS1, SENCR, LN892 and KIF25-AS1 (Figure 3D). Finally, we used online tool GEPIA(62) to explore the association between the expression of these lncRNAs and patient survival. As expected, overexpression of LN989, SENCR, LN892 and KIF25-AS1 significantly or has a trend to predict shorter survival time (Figure 3E). There were no survival plots for PROX1-AS1 in GEPIA. Moreover, higher levels of LN989, SENCR, LN892 and KIF25-AS1 tend to predict poorer survival in leukemia patients from the TCGA study using two different comparisons (Figure S6b). The Kaplan-Meier test was not performed on PROX1-AS1 due to its extremely low expression in TCGA leukemia patients (median of FPKM < 0.003). Together, these results indicate that upregulation of m^6^A-associated lncRNAs^23,24^could be a common vulnerability and prognostic factors in CML and AML patients post TKI therapy.

### LncRNA upregulation in resistant cells takes place through the enhanced RNA stability by FTO-mediated m^6^A demethylation

**Figure 4:**
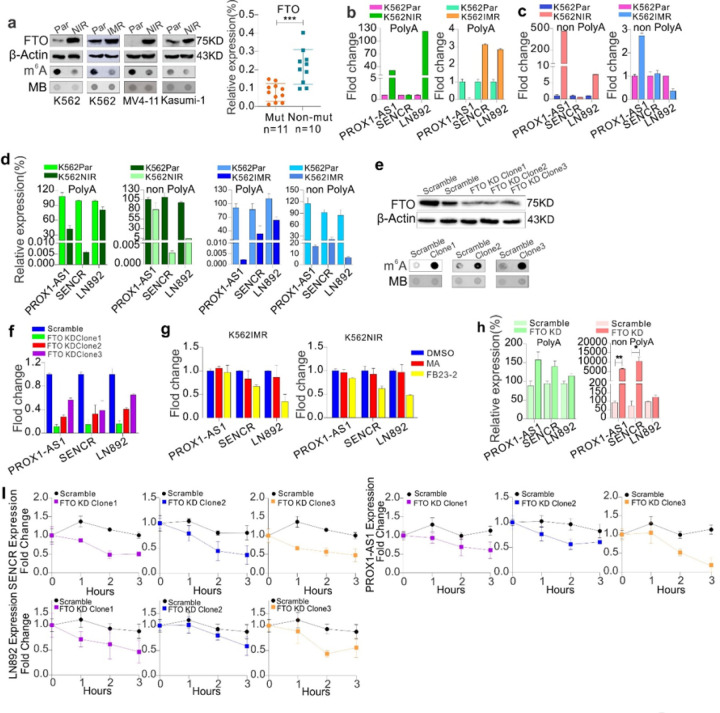
LncRNAs are partially regulated by FTO-dependent m^6^A methylation in resistant cells. **(A)** Left: Dotblotting for global m^6^A amounts (lower) and Western blot for FTO protein expression (upper) in K562, MV4–11 and Kasumi-1 cells resistant to nilotinib or imatinib. Data represent three independent experiments. The figures were made by cropping the large gel or blot images. Right: qPCR for FTO expression in resistant CML patient with/without BCL/ABL mutations. **(B-C)** m^6^A immunoprecipitation (IP) was performed in mRNA/poly-A (B) and non-poly-A RNA (C). The eluted RNA was converted to cDNA and lncRNA expression was assessed by qPCR. **(D)** qPCR for the m^6^A-IP relative expression of lncRNAs normalized by the total levels. **(E)** Dotblotting for global m^6^A methylation (lower) and Western blot for FTO protein expression (upper) in clones with FTO knockdown. Data represent three independent experiments. The figures were made by cropping the large gel or blot images. **(F)** qPCR for lncRNA expression in clones with FTO knockdown. **(G)** qPCR for lncRNA expression in resistant cells treated with meclofenamic acid (50 μM) and FB23–2 (10 μM) for 6 hours. **(H)** m^6^A IP was performed in mRNA and non-poly-A RNA in clones with FTO knockdown. The eluted RNA was converted to cDNA and expression of PROX1-AS1, SENCR, LN892, normalized by total RNA, was assessed by qPCR. **(I)** qPCR for lncRNA expression in scrambled and FTO knockdown clones treated with 5 μg/ml actinomycin-D for the indicated time points. The gene expression was normalized to GAPDH. In all qPCR, data are mean ± S.E.M. Par, parental; NIR, nilotinib resistant; IMR, imatinib resistant; KD, knockdown.

Given that m^6^A binding motifs are enriched within lncRNAs,(36) and as m^6^A methylation has been found to regulate the stability of RNA transcripts,(36,64) lncRNA upregulation in resistant cells may result from prolonged half-life of RNA transcripts, due to m^6^A demethylation. To this end, we first measured the levels of global m^6^A amounts and FTO expression, and observed a decrease of m^6^A abundance (Figure 4A, left; Figure S7a) and an increase of FTO expression (Figure 4A, left) in K562, MV4–11 and Kasumi-1 cells resistant to nilotinib or imatinib, in line with our previous reports.(36) Further, FTO levels were significantly elevated in nilotinib-treated AML patients and in CML patients non-responding to imatinib or nilotinib therapy (Figure S7b). Most notably, TKI non-responders without acquired kinase domain mutations have significantly higher FTO expression than those carrying mutations (Figure 4A, right). As lncRNAs include poly-A and non-poly A tailed, we then performed RNA IP using anti-m^6^A antibody in both poly-A and non-poly A fraction, converted the eluted RNA to cDNA and carried out qPCR with primers covering m^6^A binding sites. As shown in Figure4b and 4c, the m^6^A amounts for PROX1-AS1, SENCR and LN892 were increased without obvious changes in SENCR expression in nilotinib-resistant K562 cells when using GAPDH as internal control. For imatinib-resistant K562 cells, the amounts for PROX1-AS1 with poly-A tailed and LN892 without poly-A tailed were decreased. Because the total levels of these lncRNAs are dramatically increased in resistant versus parental cells (ref. Figure 2D, left), to accurately reflect the changes of m^6^A amount specific for these lncRNAs, we normalized the m^6^A-IP related alterations to the changes of total lncRNA levels, and observed that the m^6^A levels of PROX1-AS1, SENCR and LN892 are decreased in resistant versus parental controls, in parallel with the changes in global m^6^A content (Figure 4D). These findings suggest that m^6^A hypomethylation accounts for lncRNA upregulation in resistant cells.

To study whether FTO, a key m^6^A demethylase,(36) plays a role in lncRNA dysregulation, we infected K562 cells with scramble or FTO shRNAs (shRNA-TRCN0000183897, TRCN0000179651, TRCN0000180978) viruses, and selected TRCN0000180978 for further investigation, because TRCN0000180978 had the highest efficacy to knock down FTO (Figure S7c). Colony-forming assays revealed that FTO knockdown impairs colony potential as evidence by a decrease of colony number and size (Figure S7d). Then single clones were selected and expanded for further investigations. Firstly, knockdown of FTO and increase of m^6^A methylation were confirmed in each clone (Figure 4E; Figure S7e). Secondly, qPCR analysis disclosed that expression of PROX1-AS1, SENCR and LN892 is significantly inhibited in clones with FTO knockdown compared with scrambled controls (Figure 4F). Thirdly, in line with FTO knockdown, pharmacological inhibition of FTO by meclofenamic acid (MA)(65) and FB23–2(66) downregulated these lncRNAs in K562 cells resistant to imatinib or nilotinib (Figure 4G). Notably, no obvious changes in FTO protein expression were observed (Figure S7f) suggesting the enzymatic inhibition by MA and FB23–2; the increased downregulation of lncRNAs by FB23–2 was consistent with its higher and more selective FTO enzymatic inhibition when compared with MA.(66) To assess changes of m^6^A on individual lncRNAs, we performed m^6^A IP in total RNAs, poly-A and non-poly-A tailed RNAs in clones with scrambled and FTO knockdown. qPCR analysis revealed that m^6^A content of PROX1-AS1, SENCR, and LN892 is significantly increased when normalized to the expression of lncRNAs from total RNAs (Figure 4H). These results suggest that FTO upregulates lncRNAs through decreasing m^6^A amount of specific lncRNAs.

It has been shown that m^6^A methylation predominantly and directly decreases transcript stability.(64) To address why the m^6^A-containing lncRNAs are downregulated in FTO knockdown cells, we examined the RNA stability in FTO knockdown and control cells. Namely, we treated FTO knockdown or control clones with antinomycin-D, a transcription inhibitor.(36) The total RNA was extracted and the lncRNA levels were assessed by qPCR, which was normalized to GAPDH. As expected, the decay of lncRNAs was shortened in FTO knockdown cells compared to scrambled cells (Figure 4I). Collectively, these findings suggest that lncRNA upregulation in resistant cells is attributed to longer RNA half-life, which is mediated by FTO-dependent m^6^A demethylation.

### Genetic suppression of the FTO-lncRNA axis promotes nilotinib sensitivity, and decreases proliferation and growth of resistant cells

**Figure 5:**
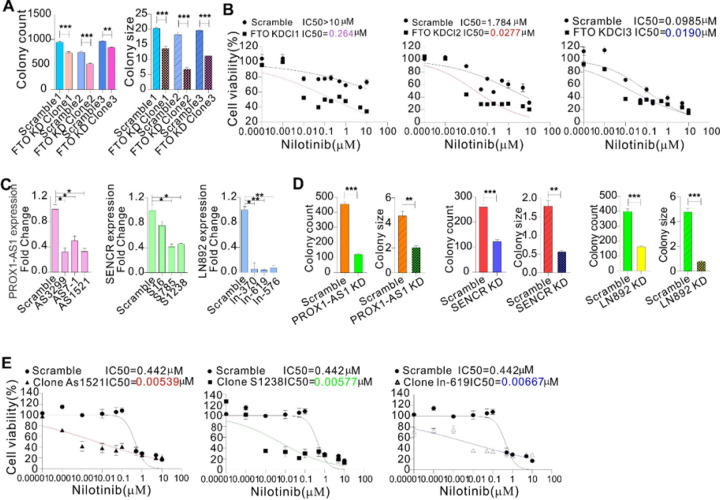
FTO-regulated lncRNAs contribute to nilotinib sensitivity. **(A)** Colony-forming assays in K562 clones with FTO knockdown. **(B)**CCK-8 assays in FTO knockdown or control clones treated with nilotinib for 48 hours. The data represent two independent experiments with 6 repeats in total. **(C)**Resistant K562 cells were infected with lncRNA virus for 48 hours, and qPCR was used to assess lncRNA expression. **(D)** Colony-forming assays in K562 resistant cells with knockdown of indicated lncRNAs. **(E)** CCK-8 assays in lncRNA knockdown or control clones treated with indicated doses of nilotinib for 48 hours. The data represent two independent experiments with 6 repeats in total. KD, knockdown. **P< 0.01, ***P < 0.001.

Having demonstrated the regulatory roles of FTO and m^6^A in lncRNA expression, we proceeded to address the impacts of lncRNAs on TKI sensitivity using K562 clones with stable FTO knockdown. We first confirmed that FTO knockdown impairs colony potential as evidence by a decrease of colony number and size compared to scrambled controls (Figure 5A); expression of lncRNAs, including LN989, LN892, LN504, PROX1-AS1, SENCR, and KIF25-AS1, was significantly decreased in cells with FTO knockdown (Figure S8a). We then examined the survival of FTO knockdown or scrambled clones upon transient exposure to nilotinib. As shown in Figure 5B, the scrambled clones had IC_50_ values to nilotinib several orders of magnitude larger than those exhibited by FTO knockdown. Although all FTO knockdown clones displayed significant and dose-dependent decreases of cell viability, the scrambled clones could proliferate at drug concentrations much larger than the IC_50_ value, in agreement with our previous findings (36) that FTO positively regulates TKI sensitivity in leukemia cells.

We have shown that lncRNA upregulation in TKI non-responding CML patients predicts worse outcomes. If lncRNAs confer increase of TKI protection to leukemia cells, then ablating lncRNAs in these cells should confer increased sensitivity to TKIs. To test this hypothesis, we knocked down LN892, PROX1-AS1 or SENCR in K562 resistant cells carrying lncRNA upregulation (Figure 5C), because these three lncRNAs carry m^6^A motifs and are highly expressed with a marked decrease of their m^6^A methylation in resistant cells. Colony assays showed that knockdown of PROX1-AS1, SENCR or LN892 reduces colony number and size (Figure 5D, Figure S8b). To test whether lncRNA abundance affects nilotinib sensitivity, we treated lncRNA knockdown clones with various concentrations of nilotinib for 72 hours. We found that knockdown of lncRNAs renders the resistant clone sensitive to nilotinib-inhibited cell proliferation, as supported by showing that the IC_50_ values in clones with PROX1-AS1 (AS1521), SENCR (S1238) or LN892 (ln-619) depletion are several orders of magnitude lower than those in scrambled (Figure 5E). Collectively, these results suggest that lncRNAs enable culture leukemia cells to better withstand nilotinib-induced cell death.

### LncRNA-fueled growth of resistant cells is linked to the active PI3K-AKT signaling pathway

The function of PROX1-AS1, SENCR or LN892 in regulating TKI resistance may be through a change in a specific gene program. To gain further insights of lncRNA-conferred resistant cell growth, we first analyzed a public database (GSE51878) using LncRNA2Target v2.0, in which SENCR was knocked down in cancer cells, to identify lncRNA downstream targets. We focused on SENCR, but not PROX1-AS1 and LN892, because the pathogenic roles of SENCR have been documented in cancer, but limited knowledge is available for PROX1-AS1 and LN892. Importantly, our findings revealed that SENCR is also subjected to regulation by FTO-dependent m^6^A hypomethylation, and affects response to TKIs *in vitro* and in patients and impacts patient survival. The signature of SENCR consisted of 663 differentially expressed genes (Supplementary table 4). Using DAVID 6.8 for functional annotation, KEGG analysis provided insights into biological processes enriched in SENCR knockdown cells, and revealed many functional pathways (Supplementary table 5), which involve 291 SENCR-regulated genes.

**Figure 6:**
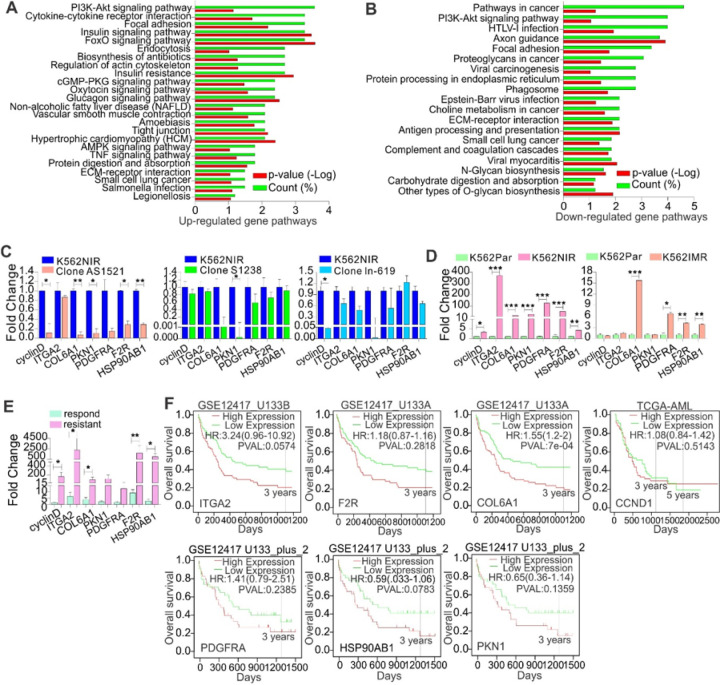
PI3K signaling pathway mediated lncRNA-sustained resistant cell growth. **(A-B)** KEGG pathway analysis identifies a network of genes related to PI3K signaling that is responsible for lncRNA-sustained cell growth. Functional pathway analysis was performed in differentially expressed genes upon SENCR knockdown using DAVID 6.8/KEGG software. Up-regulated (A) and down-regulated (B) gene pathways were determined by comparing SENCR knockdown and scrambled control cells. Count (%) means the ratio of genes involved in the pathway. **(C)** The expression of indicated genes was assessed by qPCR in K562 resistant clones with knockdown of PROX1-AS1, SENCR or LN892. **(D)** The expression of indicated genes was assessed by qPCR in K562 parental and resistant (nilotinib, imatinib) cells. **(E)** The expression of ITGA2, COL6A1, cyclin D1, PKN1, PDGFRA, F2R, and HSP90AB1 was assessed by qPCR in TKI responding and resistant CML patients. **(F)** The association of ITGA2, COL6A1, cyclin D1, PKN1, PDGFRA, F2R, and HSP90AB1 expression with overall survival (OS) in leukemia patients analyzed by the Kaplan– Meier estimate using online tool PROGgene or GEPIA2. In F, the curves (ITGA2, F2R, COL6A1, cyclin D1) were adjusted for age and cohort divided at median of gene expression. In all qPCR, data are mean ± SD.

To select the most important pathways for further investigations, we performed functional pathway analyses for all differentially expressed genes together (n = 663), or dividing them into up- (n = 337) or downregulated (n = 326) groups. All these strategies nominated the phosphatidylinositol 3-kinase (PI3K) pathway as mediating lncRNAs-sustained resistant cell growth (Figure 6A and B; Figure S9). This was especially interesting given that the PI3K signaling plays an important role in leukemia pathogenesis with limited knowledge in drug resistance.(67,68) Although 13 up- and 12 down-regulated genes were enriched in the PI3K signaling, we focused on 7 top-ranked downregulated genes (e.g., ITGA2, COL6A1, cyclin D1, PKN1, PDGFRA, F2R, HSP90AB1), because SENCR behaved as an oncogenic lncRNA in TKI resistance. Further, these genes were either downregulated to the highest levels in cells with SENCR knockdown, or well-established resistant genes and/or annotated to have a role in sustaining cancer cell survival and proliferation (Figure S9).(36,69,70) qPCR on RNAs from independent samples confirmed that, compared with scrambled controls, ITGA2, COL6A1, cyclin D1, PKN1, F2R, PDGFRA, and HSP90AB1 are downregulated in imatinib- and nilotinib-resistant K562 clones with knockdown of LN892, PROX1-AS1 or SENCR (Figure 6C). These findings place PI3K signaling as downstream of all three lnsRNAs PROX1-AS1, SENCR and LN892 in resistant cells.

Finally, the merits of the aforementioned genes within PI3K signaling warrant further insights before we could abrogate this signaling and assess the consequences to TKI resistance. We examined the expression of ITGA2, COL6A1, cyclin D1, PKN1, F2R, PDGFRA, and HSP90AB1, and found that these genes are significantly upregulated in imatinib or nilotinib-resistant versus sensitive K562 cells (Figure 6D) and in TKI resistant CML patients compared to responding counterparts (Figure 6E). To address the clinical relevance of these PI3K signaling downstream effectors that we unearthed *in vitro*, we first compared the expression of ITGA2, COL6A1, cyclin D1, PKN1, F2R, and PDGFRA among patients at a diagnosis with follow-up information available. In AML patients, a trend towards higher levels of expression of most genes was observed in the ELN risk groups intermediated and adverse (Figure S10a). In line, a trend towards higher expression of COL6A1, F2R and ITGA2 at diagnosis in AML patients that did not achieve CR after induction chemotherapy was observed (Figure S10b). In line, a trend towards higher expression of COL6A1, F2R and ITGA2 was seen in blast phase CML compared to samples at initial diagnosis (Figure S10c). Notably, the predictable capacity is heterogeneous and not robust, which warranty further verification by co-founding with multiple factors, including blast percentage, genetic mutations, age and sex, in a larger cohorts of patients. We then analyzed public datasets (GSE12417 or TCGA-AML) to examine whether expression of these targets is associated with patient survival. We found that upregulation of ITGA2, COL6A1, cyclin D1/CCND1, PKN1, PDGFRA and F2R genes predicts or at least has trends toward worse outcomes (Figure 6F). This result was further verified by the findings from GEPIA-mediated analysis (Figure S10d). Collectively, these results provide compelling evidence that key components of the PI3K-AKT signaling are responsible for lncRNA-sustained TKI resistant cell growth.

### TKI resistant cells are sensitive to PIK3 inhibitor alpelisib *in vitro* and *in vivo*

**Figure 7:**
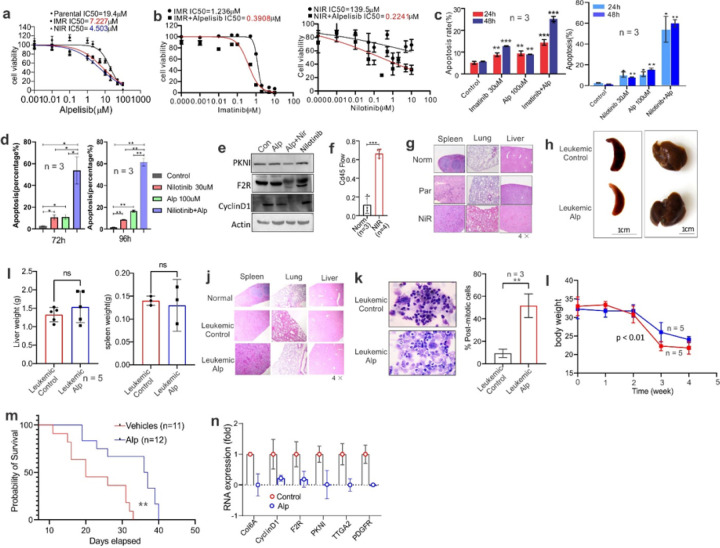
Inactivation of PIK3 signaling by alpelisib in TKI resistant cells achieves leukemia remission *in vitro* and *in vivo*. **(A)** CCK-8 assays in K562 parental and resistant cells to imatinib (IMR) or nilotinib (NIR) with various concentrations of alpelisib for 72 hours. **(B)** CCK-8 assays in K562 IMR or NIR resistant cells with various concentrations of imatinib or nilotinib with/without alpelisib (100 uM) for 72 hours. **(C-E)** IMR or NIR resistant K562 were treated with alpelisib (Alp; 100 uM), imatinib (30 uM) or nilotinib (30 uM) alone or combination for the indicated time points. The treated cells were subjected to flow cytometry (**C**, **D**) for cell apoptosis or Western blot (72 hours; **E**). The figures were made by cropping the large gel or blot images. Graphs are the quantification of apoptotic cells presented as percentage. **(F-G)** About 0.5 x 10^6^ nilotinib resistant K562 cells were injected through tail-vein into sub-lethally irradiated NSGS mice (n = 5 mice/group). The BM cells were isolated for (**F**) FACS analysis of the engrafted recipient BM cells (stained by CD44 antibody) from the representative disease mice (5 weeks after cell injection). Graphs are the quantification of CD45+ cells (folds). (**G**) H&E staining of lung, liver or spleen sections (original magnification × 4) from healthy (norm) or leukemic mice bearing parental (Par) or nilotinib resistant (NIR) cells. **(H-N)** About 0.5 × 10^6^ BM cells isolated from F were injected via the tail-vein into sub-lethally irradiated NSGS mice (second recipient). Five days after cell injection, the mice were randomly grouped and treated with Alp. **(H-I)** Representative external views of the spleens from the leukemic mice (**H**) and the weight of spleen and liver (**I**). **(J)** H&E staining of lung, liver or spleen sections (original magnification × 4) from healthy (normal), leukemic mice treated with vehicles (control) or Alp (n = 3) (magnification × 4). **(K)** Representative images of Wright-Giemsa-stained cytospins of BM cells from leukemic mice (left; magnification × 40) and graph (right) shows quantification of post-mitotic cells from (**E**). **(L)** Graphs show the changes in body weight of vehicles or alp-treated mice (n = 10). **(M)** Effects of alpelisib on survival of leukemia-bearing mice were determined by the Kaplan-Meier estimate (log-rank test). **(N)** BM cells isolated from treated mice were subjected to qPCR for the indicated lncRNA targets using primers of human genes. In **A-B**, the data represent two independent experiments with 8 repeats in total; in **C**, **D** and **E**, data represent three independent experiments. Note, the survival time is from the start of leukemia cell injection. Data are shown as mean values ± SD, *P < 0.05; **P < 0.01; ***P < 0.001.

Having shown that PI3K signaling is essential for leukemia TKI resistance, we reasoned that pharmacological targeting of PI3K signaling could override resistant cells. To test this, we selected alpelisib, an FDA approved first PI3K inhibitor for breast cancer, which has not been tested in leukemia. When parental or resistant K562 cells were treated with alpelisib *in vitro* for 72 hours, the growth of drug-resistant cells was significantly inhibited by alpelisib in a dose-dependent manner with IC_50_ values in parental cells that are 2.7-fold or 4.3-fold higher than that in IMR or NIR cells (Figure 7A). The lower IC_50_ values in resistant cells indicated the higher activation of PI3K signaling and thus more sensitive to alpelisib treatment. Further, the addition of alpelisib lowered the IC50 of imatinib by 3.5 folds and the IC50 of nilotinib by 625 fold in imatinib- or nilotinib-resistant cells (Figure 7B). We then treated resistant cells with alpelisib alone or in combination with imatinib or nilotinib for 24, 48, 72 or 96 hours. Although alpelisib, imatinib or nilotinib as single drugs marginally slowed down resistant cell growth, their combination resulted in more pronounced impairment of cell proliferation, as demonstrated by the highest rate of cell apoptosis (Figure 7C and D). Mechanistically, combination of alpelisib with nilotinib led to more reduction of PIK3 signaling mediators, such as PKNI, F2R and Cyclin D1 (Figure 7E). These results imply that PIK3 signaling is a therapeutic target for overcoming TKI resistance.

To further determine the clinical implications of PI3K signaling in TKI resistance, we established a CML mouse model by injecting 0.5 x 10^6^ nilotinib resistant K562 cells via the tail-vein into sublethally irradiated (2.5 Gy) triple transgenic NSG-SGM3 (NSGS) mice (male). The successful engraftment of CML cells was verified by the identification of human CD45+ cells in mouse bone marrow (BM) cells (Figure 7F) and the infiltration of CML K562 cells to mouse organs (e.g., liver, lung, spleen) (Figure 7G). Then the BM cells (0.5×x 10^6^) were isolated from mice bearing K562 resistant cells and injected into the second recipient NSGS mice 4 hours after sublethal irradiation. These leukemic mice were randomly grouped and sequentially given 30 or 50 mg/kg of alpelisib(71) in PEG400 and saline (ratio 15:38:47) intraperitoneally twice a week for a total of eight doses. The leukemic mice injected with only vehicle served as controls. Although the size and weight of spleen and liver did not show significant difference (Figure 7H and I), H&E staining revealed that, compared to the alpelisib-treated mice, the vehicle group displayed an increased infiltration of leukemic cells into the spleens, lungs and livers of recipients, leading to considerable damage to these organs (Figure 7J). The BM histopathology from alpelisib-treated mice identified more differentiated cells containing metamyelocytes, bands, and segmented neutrophils compared to vehicle-treated mice (Figure 7K). Alpelisib-treatment significantly slowed the decrease of body weight (Figure 7L), importantly, increased the survival time of leukemic mice (Figure 7M). No toxicity was observed for the tested drug dosage and schedule because we did not see any evident change in capability of moving and getting food and water when comparing alpelisib-treated mice to vehicle groups. Mechanistically, alpelisib administration in mice remarkably downregulated PI3K signaling mediators, including ITGA2, COL6A1, cyclin D1/CCND1, PKN1, PDGFRA or F2R (Figure 7N). Of note, the leukemic mice were bearing human CML K562 cells, thus we used primers for human, but not mouse, genes to detect the aforementioned genes. This approach could verify the limited toxicity of alpelisib to leukemic mice and demonstrate the specificity of CML cell killing by alpelisib *in vivo*. Together, these findings provide critical means of targeting PIK3-AKT signaling to treat patients with refractory leukemia post TKI therapy.

## Discussion

Treatment with TKIs often leads to development of resistance that has been a major hurdle to successful cancer treatment. However, mechanisms that generate and sustain drug-resistant cell populations as well as molecular predictors for prognosis and drug response are still unclear, particularly for resistant patient subpopulation without acquired TK kinase domain mutations. By dissecting the impact of a dynamic m^6^A methylome on TKI sensitivity, we demonstrate that the m^6^A-regulated lncRNAs are deregulated molecules whose overexpression in TKI resistant cell lines and in patients with inadequate response to TKI therapy is important not only for resistant cell proliferation, but also for insensitivity to TKI treatment and patient survival. We also show that the FTO-dependent m^6^A demethylation can regulate lncRNAs by increasing their RNA stability, and lncRNA-activated PI3K signaling renders insensitivity to TKIs and sustain resistant cell growth. Further, we observe that, among refractory or relapsed population, FTO levels are significantly higher in patients without kinase mutations compared to those with mutations; lncRNA upregulation and the activated PI3K signaling detected in patients at a diagnosis is associated with the follow-up bad drug response or unfavorable outcomes. Our findings add a new layer to the complexity of mechanisms regulating leukemia cell fate under TKI selection, and raise the possibility that the m^6^A-regulated lncRNAs represents a new non-genetic factor to affect the development and maintenance of TKI resistance; our discoveries identify a promising therapeutic target, FTO, for specifically, the most challenging patient subpopulations who are TKI non-responders/relapsed but do not carry the acquired BCR::ABL1 mutations; our results uncover a strong predictor, m^6^A-regulated lncRNA-PI3K axis, for poorer prognosis and failure in drug response in cancers.

Acquired resistance to TKIs is commonly attributed to genetic mechanisms.(8, 72) However, evidence is emerging that the development of drug resistance is accomplished without genetic alterations.(73–75) Targeting epigenetic machinery has become a major thrust in the development of new therapeutic strategies; unfortunately, these strategies have yielded mixed results in clinical trials. One barrier to progress in harnessing epigenetic therapies to overcome drug resistance is the limited understanding of how cancer cells can rapidly escape TKI killing. We recently demonstrated(36) that a dynamic and reversible m^6^A methylome represents a non-genetic driver of a TKI-tolerance state in heterogeneous leukemia cells, yet the molecular mechanisms by which the TKI-altered m^6^A methylome affects drug sensitivity are incompletely understood. Because transcriptome-wide m^6^A sequencing disclosed that most changes of m^6^A peaks in resistant cells come from lncRNAs,(36) this study was designed to test the hypothesis that m^6^A-associated lncRNAs could be additional key non-genetic factor to fuel and sustain resistant cell growth. As expected, we identified and validated many annotated lncRNAs, which are differentially expressed with an obvious change of m^6^A content in TKI resistant cell lines and non-responding leukemia patients.

LncRNA defects have been found to affect drug resistance,(76) but it is unclear whether an interaction between m^6^A and lncRNAs exists in leukemia resistance against TKIs. To test whether m^6^A-assicated lncRNAs play a role in TKI response, we initially examine their role in leukemia patients. We observed that these lncRNAs, including PROX1-AS1, SENCR or LN892, are elevated in leukemia patients vs normal donors, and in CML patients not responding to TKIs or with blast crisis disease compared with TKI responders or with chronic disease. We further observed that patients with higher lncRNA expression at least have a trend toward worse prognosis than those with lower levels. These findings support PROX1-AS1, SENCR or LN892 as prognostic biomarkers in leukemia therapy. We next knocked down the upregulated PROX1-AS1, SENCR or LN892 in resistant cells, and found that lncRNA knockdown inhibits resistant cell growth, and sensitizes nilotinib resistant cells to nilotinib killing. These findings suggest that lncRNA upregulation is required, at least partially, to support resistant cell growth and to preserve TKI insensitivity.

It is increasingly appreciated that lncRNAs are key regulators in cancer pathogenesis and drug resistance.(38–46) However, they are not the direct contributors, but determine cancer cell fate through up- or down-regulating cancer cell determinant genes. Further, very few studies have been found to address the role of PROX1-AS1, SENCR or LN892 in drug resistance. By analyzing a gene expression profile resulting from SENCR knockdown, we identified a downstream mediator, PI3K signaling, which represents a central regulatory node controlling cell proliferation and apoptosis, and plays a critical role in leukemia drug resistance.(77) The key genes (i.e., ITGA2, COL6A1, cyclin D1, PKN1, PDGFRA and F2R), which are changed the most and likely involved in the PI3K pathway, could be downstream targets of PROX1-AS1, SENCR or LN892, because knockdown of these lncRNAs impairs the aforementioned target expression. These lncRNA-regulated genes are overexpressed in TKI resistant cells and in imatinib or nilotinib non-responding patients compared to good responders. Leukemia patients with overexpression of ITGA2, COL6A1, cyclin D1, PKN1, PDGFRA or F2R predicts or has trends toward worse prognosis. However, it will be interesting to see in the future whether these PI3K targets are prognostic biomarkers in larger cohort of patients, which should also be justified by age, mutation status, sex, disease stages and induction pre-treatment.

The molecular basis of lncRNA dysregulation in resistant leukemia is still obscure. We focused on m^6^A methylation in regulating lncRNAs, because 1) a dynamic m^6^A methylome represents a bona fide defense mechanism in developing TKI resistance; 2) half of the differentially expressed lncRNAs have m^6^A motifs; 3) m^6^A reduction and lncRNA upregulation co-exist in resistant cells; 4) the abundance of m^6^A plays a key role in RNA stability; and 5) the transcriptional repression by lncRNAs is enhanced by m^6^A methylation.(47, 48) Our studies revealed that, in parallel with global m^6^A hypomethylation, m^6^A content specific for upregulated lncRNAs, like PROX1-AS1, SENCR or LN892, is significantly decreased in resistant cells. Given that upregulation of these lncRNAs affects TKI sensitivity and patient survival, these results support a crosstalk between m^6^A methylome and lncRNA pathway in TKI resistance. Mechanistically, FTO serves as a new lncRNA regulator through FTO-dependent m^6^A hypomethylation in leukemia resistant cells. In support of this possibility was the downregulation of PROX1-AS1, SENCR or LN892 and the reduction of their m^6^A levels when FTO is inactivated by either gene knockdown or inhibitor treatment. This is followed by an increased RNA half-life of lncRNA transcripts. These findings provide an alternative explanation for why many lncRNAs are differentially expressed in TKI resistant cells. It would also be important to know whether all identified lncRNAs are direct targets of FTO and whether FTO dependent m^6^A affects RNA stability of all these targets.

While TKIs (*e.g.*, imatinib, nilotinib) have revolutionized leukemia treatment,(2–5) TKI don’t cure all leukemia patients, due to the development of TKI resistance. Further, a subpopulation of patients (30– 50% in CML) who achieve complete molecular remission, must take TKIs for the rest of their lives, which can lead to severe side effects and substantial financial burden. Thus, the development of novel therapies to overcome resistance are major goals in the field. After demonstrating the key role of lncRNA-PI3K axis in regulating TKI resistance, we explored the therapeutic potential of PI3K inhibitor alpelisib in treating patients with resistant CML. Notably, PI3K signaling is an important downstream mediator in BCR-ABL-driven leukemia, but alpelisib has not been tested, particularly, for refractory CML patients. We showed that alpelisib sensitizes imatinib or nilotinib resistant cells to imatinib or nilotinib-induced cell death, and its combination with imatinib or nilotinib results in more pronounced cells apoptosis *in vitro*. Most importantly, alpelisib-therapy in NSGS mice bearing nilotinib-resistant K562 cells significantly reduces leukemia burden and increases the survival time of leukemic mice, mechanistically through suppression of PI3K signaling mediators like F2R, CyclinD1 and PDGFR. These findings support alpelisib as a novel therapeutic reagent for TKI resistant leukemia, opening up promising new avenues to treat patients with recurrent disease.

In summary, this study discovers a critical role that m^6^A-regulated lncRNAs play in sustaining resistant growth, maintaining resistant phenotypes and in promoting TKI insensitivity *in vitro* and in CML patients receiving TKI therapy. It reveals a novel mechanism, the FTO-m^6^A-lncRNAs axis, to explain why lncRNAs are elevated in TKI resistant cells, and also a pathway, lncRNA-regulated PI3K signaling, to answer the question of how lncRNA dysregulation affects TKI resistance. Given that m^6^A-regulated lncRNAs affect TKI response and survival of leukemia patients, it will be interesting to see in the future whether these hitherto underappreciated roles for m^6^A-lncRNAs-PI3K cascade in anti-cancer resistance could serve as a foundation to design therapeutics that might overcome TKI resistance for this devastating cancer.

### Limitations of the study

The study has some limitations. First, we revealed a dynamic RNA m6A methylome(36) and identified a set of lncRNAs (see Fig. 3), which are shared by AML and CML cells post TKI treatment, the current study used only CML as readout model to get further insights to cancer drug resistance. The role of m6A-regulated lncRNAs in AML might be challenging to explore due to the “7 + 3” regimen before TKI therapies. Second, the identification and characterization of lncRNA abnormalities was limited to relatively small cohorts of patients at the diagnostic stage, blast crisis phase or TKI non-responders. The use of the tested lncRNAs as molecular predictors for drug resistance and poorer prognosis is required to be validated in larger cohorts of patients from the multiple centers. Third, our animal models to pharmacologically test inhibitors (e.g., alpelisib) for the lncRNA-PI3K axis are limited to leukemic mice bearing resistant cell lines. The patient-derived xenograft (PDX) models bearing nilotinib resistant CML patient blasts can further explore the clinical implications of alpelisib in overcoming leukemia TKI resistance. Fourth, we only targeted PI3K signaling, one of multiple pathways controlled by dysregulated lncRNAs in resistant cells, to overcome TKI resistance. Given the key roles of high m6A-enriched lncRNA expression in resistant patient cells, future studies can further exploit the therapeutic potentials of targeting lncRNAs themselves (*e.g.*, SENCR, PROX1-AS1, LN892), more challenging due to *in vivo* delivery approaches but with higher efficacy due to multiple downstream effector pathways.

## Data Availability

All relevant data supporting the key findings of this study were available within the article and its Supplementary Information files or from the corresponding authors upon reasonable request. Partial data were obtained from “Public data analysis” including GEO datasets (GSE80481; GSE51878).
